# Supplementary material for: Periodic Implementation of the Random Phase Approximation with Numerical Atomic Orbitals and Dual Reciprocal Space Grids
Source: J Chem Theory Comput. 2025 Sep 18;21(19):9347–63. doi: 10.1021/acs.jctc.5c00751 (PMC12529921; doi:10.1021/acs.jctc.5c00751)
Supplement: Supplementary file 1 [file ct5c00751_si_001.pdf]

Supporting Information to "Periodic implementation of the random phase approximation with numerical atomic orbitals and dual reciprocal space grids"

Edoardo Spadetto,[*,†,‡] Pier Herman Theodoor Philipsen,[*,‡] Arno Förster,[*,†] and Lucas Visscher[*,†]

†*Theoretical Chemistry, Vrije Universiteit, De Boelelaan 1108, 1081 HZ Amsterdam, The Netherlands*

‡*Software for Chemistry and Materials NV, NL, 1081HV, Amsterdam, The Netherlands*

E-mail: e.spadetto@vu.nl; philipsen@scm.com; a.t.l.foerster@vu.nl; l.visscher@vu.nl

Contents

S1 Comparison between molecules and periodic system **2**

S1.1 Results: Noble gas chains 4

S2 3D simple cubic Noble Gases topologies **5**

S3 MgO-CO energies **7**

S3.1 Monolayer MgO-CO adsorption energy 8

S3.2 MgO-CO blayer calculations 9

S3.2.1 $\boldsymbol{k}$-mesh convergence 9

S3.2.2 Coverage-dependence 10
S3.3 Convergence with the number of MgO layers 12
S3.4 SCF energies for CO adsorption on the 4-layer MgO slab 12
References **12**

S1 Comparison between molecules and periodic system

Consider a 1D system with lattice constant a and basis set $\{\chi_\mu(k_i)\}$. The basis functions are

$$\chi_\mu(\boldsymbol{r}, k_i) = \sum_n e^{ik_i \cdot na}\chi_\mu(\boldsymbol{r} - na) \tag{1}$$

Splitting the Bloch sum to consider the distance between two primitive cells we get

$$\chi_\mu(\boldsymbol{r}, k_i) = \sum_n e^{ik_i 2na}\chi_\mu(\boldsymbol{r} - 2na) + e^{ik_i(2n+1)a}\chi_\mu(\boldsymbol{r} - (2n+1)a) \tag{2}$$

$$= \sum_n e^{ik_i n(2a)}\chi_{\mu'}(\boldsymbol{r} - 2an) + e^{ik_i a}e^{ik_i n(2a)}\chi_{\mu''}(\boldsymbol{r} - 2an) \tag{3}$$

$$= \chi_{\mu'}(\boldsymbol{r}, k_i) + e^{ik_i a}\chi_{\mu''}(\boldsymbol{r}, k_i) \tag{4}$$

Where the indices μ' and μ'' and removal of the translation factor a, comes from substituting with basis functions from the system with two supercells.

This proves that every Bloch's sum basis function of the single primitive cell can be rewritten with the basis set from the system with a doubled primitive cell. The inverse however is not true, because in principle the 2 supercell system can break translational symmetry within the two unit cells. The case of a single unit cell is more restrictive because the next unit cell is forced to respect translational invariance as an ansatz. Nevertheless,

even though a larger Hilbert space can be represented with the two supercell case, the translational invariance of one single cell should be respected by any physical solution..

Going from the single to the double unit cell case, the size of the first Brillouin zone of the system halves,

$$
\begin{aligned}
&\chi_\mu(k_i = k_j + \frac{\pi}{a}) = \chi_{\mu'}(\boldsymbol{r}, k_i) + e^{ik_i a}\chi_{\mu''}(\boldsymbol{r}, k_i) \qquad (5)\\
&= \sum_n \left(e^{ik_i 2na}\chi_{\mu'}(\boldsymbol{r} - 2an) + e^{ik_i a}e^{ik_i 2na}\chi_{\mu''}(\boldsymbol{r} - 2an)\right)\\
&= \sum_n \left(e^{i(k_j+\frac{\pi}{a})2na}\chi_{\mu'}(\boldsymbol{r} - 2an) + e^{i(k_j+\frac{\pi}{a})a}e^{i(k_j+\frac{\pi}{a}))2na}\chi_{\mu''}(\boldsymbol{r} - 2an)\right)\\
&= e^{2\pi i}\sum_n \left(e^{ik_j 2na}\chi_{\mu'}(\boldsymbol{r} - 2an) + e^{i(k_j+\frac{\pi}{a})a}e^{ik_j 2na}\chi_{\mu''}(\boldsymbol{r} - 2an)\right)\\
&= \sum_n \left(e^{ik_j 2na}\chi_{\mu'}(\boldsymbol{r} - 2an) - e^{ik_j a}e^{ik_j 2na}\chi_{\mu''}(\boldsymbol{r} - 2an)\right)\\
&= \chi_{\mu'}(\boldsymbol{r}, k_j) - e^{ik_j a}\chi_{\mu''}(\boldsymbol{r}, k_j)\ .
\end{aligned}
$$

This equation shows that if we consider any basis function at a certain $\boldsymbol{k}$ point in the first Brillouin zone, from the single unit cell system we can rewrite it using functions from the double cell case, with the same, or with a $\boldsymbol{k}$-coordinate translated by half of the first Brillouin zone. This means, that now the Brillouin zone has dimension π/a and not as the 1 supercell case $2\pi/a$. Together, these two arguments are valid for an arbitrary number of considered primitive cells. This proves that as a limit, even an infinite-size molecule consisting of an infinite number of primitive cells evaluated only at the Γ-point, will give the same results as a periodic calculation with a converged $\boldsymbol{k}$-grid and a single unit cell.

Clearly, this infinite molecule calculation cannot be evaluated. In practice, we extrapolate RPA correlation energy for molecules of increasing size to the infinite length limit, with the assumption that the effects coming from the different boundary conditions would be local.

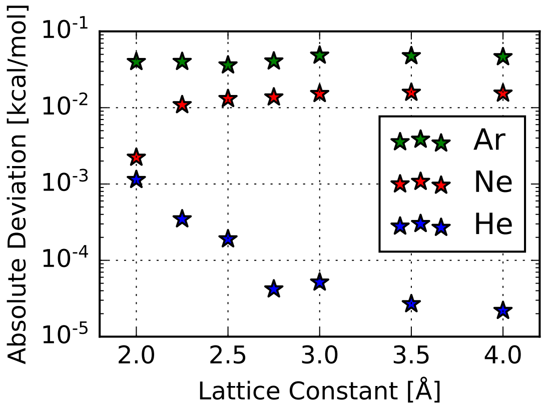

Figure S1: Absolute deviation between extrapolated molecular RPA correlation energy per atom for an infinite chain of noble gas atoms, compared with periodic result.

S1.1 Results: Noble gas chains

To prove the correctness of this periodic code we compared it with a molecular implementation[1] for noble gas chains. The boundary effects are removed from the molecular calculation by fitting the total RPA Correlation linearly for multiple lengths with

$$E_{corr}^{RPA}(N) = aN + b \; . \tag{6}$$

This way, the parameter a represents the correlation energy per unit cell without boundary effects. Instead, the periodic calculation is converged directly using a regular K_G/Q_G of 40 $\boldsymbol{k}$-points. Calculations are performed with the TZ2P basis set.[2]

S2 3D simple cubic Noble Gases topologies

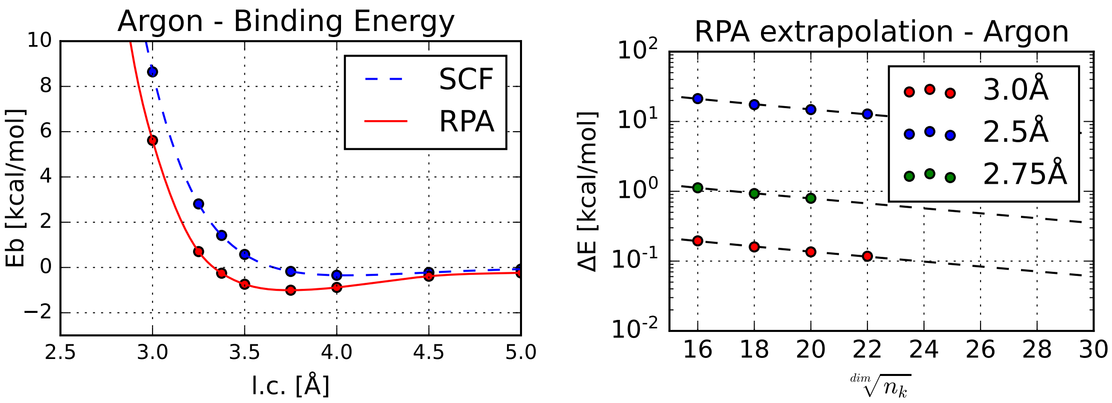

Figure S2: Left: RPA@PBE binding energy of Argon in a simple cubic lattice structure. Right: Extrapolation of RPA@PBE binding energies of Argon in a simple cubic lattice structure to the infinite $\boldsymbol{k}$-grid limit for different lattice constants.

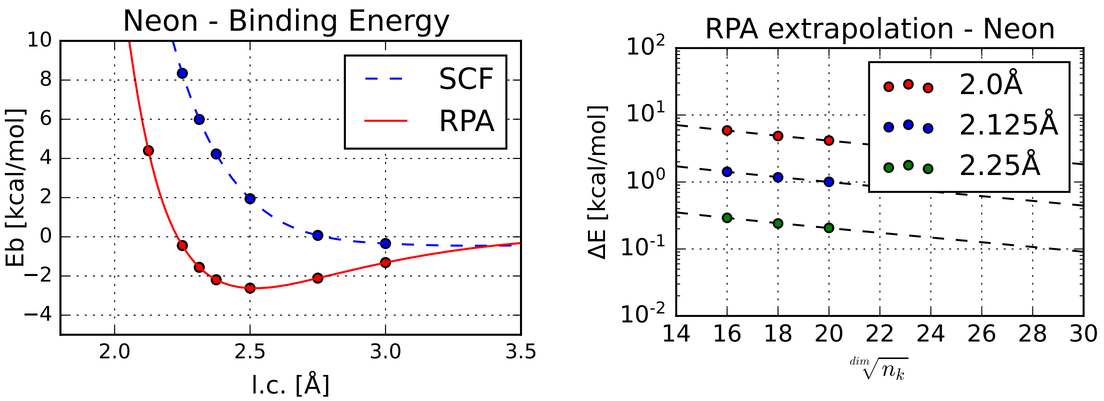

Figure S3: Left: RPA@PBE binding energy of Neon in a simple cubic lattice structure. Right: Extrapolation of RPA@PBE binding energies of Neon in a simple cubic lattice structure to the infinite $\boldsymbol{k}$-grid limit for different lattice constants.

Table S1: Equilibrium lattice constants for simple cubic Neon and Argon crystals with TZ2P basis set

	SCF [Å]	RPA[Å]
Ne	3.34	2.43
Ar	4.06	3.74

As done by Kresse and coworkers for FCC crystals,[3] we here calculated RPA@PBE binding energy curves of noble gas simple cubic lattices, highlighting how adopting the RPA cor-

rection describes the stability valley prescribed by van der Waals theory. The calculations involving Argon and Neon crystals can be seen in the left panel ofS2 and the left panel of S3 respectively. For the tightest lattice constants E_{corr}^{RPA} has been extrapolated through a $a\frac{1}{\sqrt[dim]{n_k}} + b$ fit.[4] For the larger lattice constants, no extrapolation was required to obtain converged results. Details about the correctness of the extrapolation can be seen in the right panels of figures S2 and S3, where we report the absolute deviation of the RPA correlation energies with respect to the extrapolated value for a few lattice constants. The detailed prospect of extrapolated and non-extrapolated lattice constants can be found in tables S2, S3. Table S1 shows the equilibrium lattice constants which are obtained as the minima of the binding energy curves shown in the left panels of Figs. S2 and S3.

Table S2: RPA@PBE binding energies of Argon in a simple cubic lattice. The table shows whether a lattice constant has been extrapolated to the infinite $\boldsymbol{k}$-point limit, the lattice constant, the number of points in the regular K_G/Q_G per dimension, the number of frequencies used, and the energy deviation of the extrapolated results with respect to the one obtained with the highest accuracy.

Extrapolated	l.c.	$\sqrt[\text{ndim}]{n_k}$	n_ω	ΔE
True	2.50	[16, 18, 20, 22]	32.00	22.71
True	2.75	[16, 18, 20]	32.00	1.31
True	3.00	[16, 18, 20, 22]	32.00	0.20
False	3.25	[16, 18]	32.00	0.00
False	3.38	[16, 18]	32.00	0.00
False	3.50	[16, 18]	32.00	0.00
False	3.75	[16]	32.00	0.00
False	4.00	[18]	32.00	0.00
False	4.50	[14]	32.00	0.00
False	5.00	[16]	32.00	0.00
False	5.50	[14]	32.00	0.00
False	8.00	[14]	32.00	0.00

Table S3: RPA@PBE binding energies of Neon in a simple cubic lattice. The table shows whether a lattice constant has been extrapolated to the infinite $\boldsymbol{k}$-point limit, the lattice constant, the number of points in the regular K_G/Q_G per dimension, the number of frequencies used, and the energy deviation of the extrapolated results with respect to the one obtained with the highest accuracy.

Extrapolated	l.c.	$\sqrt[\mathrm{ndim}]{n_k}$	n_ω	ΔE
True	1.50	[16, 18, 20]	32.00	1298.16
True	2.00	[16, 18, 20]	32.00	6.92
True	2.13	[16, 18, 20]	32.00	1.67
True	2.25	[16, 18, 20]	32.00	0.34
True	2.31	[16, 18, 20]	32.00	0.18
True	2.38	[16, 18, 20]	32.00	0.11
True	2.50	[16, 18, 20]	32.00	0.07
True	2.75	[16, 18, 20]	32.00	0.04
True	3.00	[16, 18, 20]	32.00	0.03
True	3.50	[8, 10, 12, 14]	32.00	0.02
False	4.00	[18]	32.00	0.00
False	4.50	[14]	32.00	0.00
False	5.00	[18]	32.00	0.00
False	5.50	[14]	32.00	0.00
False	10.00	[20]	32.00	0.00

S3 MgO-CO energies

Here we report details of the results obtained for MgO-CO adsorption for different basis sets, functionals, and coverage.

S3.1 Monolayer MgO-CO adsorption energy

Table S4: Absolute RPA@PBE energies for monolayer MgO, CO, and the corresponding dimer for different $\boldsymbol{k}$-meshes and decay parameters r_0, calculated with the DZP basis set. All values are in kcal/mol. The monomer energies are counterpoise corrected.

	$r_0 = 0.3R$			$r_0 = 0.7R$			$r_0 = 0.7R$		
$\sqrt{N_{\boldsymbol{k}}}$	MgO-CO	MgO	CO	MgO-CO	MgO	CO	MgO-CO	MgO	CO
5	-797.71	-415.34	-374.22	-796.65	-414.67	-373.71	-796.89	-414.98	-373.91
7	-797.49	-415.44	-374.01	-796.78	-415.03	-373.76	-797.01	-415.19	-373.86
9	-797.29	-415.28	-374.01	-796.92	-415.08	-373.89	-797.05	-415.16	-373.94
11	-797.21	-415.33	-373.90	-797.00	-415.22	-373.84	-797.07	-415.26	-373.86
13	-797.13	-415.27	-373.89	-797.00	-415.21	-373.86	-797.05	-415.23	-373.87
15	-797.10	-415.26	-373.88	-797.02	-415.22	-373.86	-797.05	-415.23	-373.87
17	-797.09	-415.29	-373.85	-797.04	-415.26	-373.84	-797.06	-415.27	-373.87

Table S5: Conterpoise-corrected RPA contribution to the adsorption energy of CO on monolayer MgO for different $\boldsymbol{k}$-meshes and decay parameters r_0, calculated with the DZP basis set. All values are in kcal/mol.

$\sqrt{N_{\boldsymbol{k}}}$	$E_c^{ads}(0.3)$	$E_c^{ads}(0.5)$	$E_c^{ads}(0.7)$
5	-8.15	-8.27	-8.00
7	-8.04	-7.99	-7.95
9	-8.00	-7.96	-7.96
11	-7.98	-7.94	-7.95
13	-7.96	-7.94	-7.94
15	-7.96	-7.94	-7.94
17	-7.95	-7.94	-7.94

Table S6: Absolute RPA@PBE energies for monolayer MgO, CO, and the corresponding dimer for different $\boldsymbol{k}$-meshes and decay parameters r_0, calculated with the TZ2P basis set. All values are in kcal/mol. The monomer energies are counterpoise corrected.

	$r_0 = 0.3R$			$r_0 = 0.7R$			$r_0 = 0.7R$		
$\sqrt{N_{\boldsymbol{k}}}$	MgO-CO	MgO	CO	MgO-CO	MgO	CO	MgO-CO	MgO	CO
5	-797.71	-415.34	-374.22	-796.65	-414.67	-373.71	-796.89	-414.98	-373.91
7	-797.49	-415.44	-374.01	-796.78	-415.03	-373.76	-797.01	-415.19	-373.86
9	-797.29	-415.28	-374.01	-796.92	-415.08	-373.89	-797.05	-415.16	-373.94
11	-797.21	-415.33	-373.90	-797.00	-415.22	-373.84	-797.07	-415.26	-373.86
13	-797.13	-415.27	-373.89	-797.00	-415.21	-373.86	-797.05	-415.23	-373.87
15	-797.10	-415.26	-373.88	-797.02	-415.22	-373.86	-797.05	-415.23	-373.87
17	-797.09	-415.29	-373.85	-797.04	-415.26	-373.84	-797.06	-415.27	-373.87

Table S7: Conterpoise-corrected RPA contribution to the adsorption energy of CO on monolayer MgO for different $\boldsymbol{k}$-meshes and decay parameters r_0, calculated with the TZ2P basis set. All values are in kcal/mol.

$\sqrt{N_{\boldsymbol{k}}}$	$E_c^{ads}(0.3)$	$E_c^{ads}(0.5)$	$E_c^{ads}(0.7)$
5	-8.15	-8.27	-8.00
7	-8.04	-7.99	-7.95
9	-8.00	-7.96	-7.96
1	-7.98	-7.94	-7.95
1	-7.96	-7.94	-7.94
1	-7.96	-7.94	-7.94
1	-7.95	-7.94	-7.94

S3.2 MgO-CO blayer calculations

S3.2.1 $\boldsymbol{k}$-mesh convergence

Table S8: Absolute RPA@PBE energies for bilayer MgO, CO, and the corresponding dimer as well as the corresponding contribution to the adsorption energy for different $\boldsymbol{k}$-meshes, calculated with the DZP basis set and the T1 auxiliary basis set. All values are in kcal/mol.

$\sqrt{N_{\boldsymbol{k}}}$	MgO-CO	MgO	CO	E_c^{ads}
7	−1033.02	−316.19	−710.17	−6.66
9	−1033.20	−316.23	−710.36	−6.61
11	−1033.29	−316.24	−710.46	−6.59
13	−1033.36	−316.24	−710.53	−6.59
15	−1033.37	−316.24	−710.55	−6.59

Table S9: Absolute RPA@PBE energies for bilayer MgO, CO, and the corresponding dimer as well as the corresponding contribution to the adsorption energy for different $\boldsymbol{k}$-meshes, calculated with the DZP basis set and the T2 auxiliary basis set. All values are in kcal/mol.

$\sqrt{N_{\boldsymbol{k}}}$	MgO-CO	MgO	CO	E_c^{ads}
5	−1032.26	−316.06	−709.34	−6.86
7	−1033.03	−316.19	−710.19	−6.65
9	−1033.21	−316.23	−710.38	−6.60
11	−1033.31	−316.24	−710.48	−6.59
13	−1033.37	−316.24	−710.55	−6.58
15	−1033.39	−316.24	−710.56	−6.58

Table S10: Absolute RPA@PBE energies for bilayer MgO, CO, and the corresponding dimer as well as the corresponding contribution to the adsorption energy for different $\boldsymbol{k}$-meshes, calculated with the TZ2P basis set and the T2 auxiliary basis set. All values are in kcal/mol.

$\sqrt{N_{\boldsymbol{k}}}$	MgO-CO	MgO	CO	E_c^{ads}
5	−1220.17	−374.07	−838.18	−7.93
7	−1220.79	−374.21	−838.86	−7.72
9	−1218.62	−374.07	−836.87	−7.67
11	−1218.69	−374.04	−836.98	−7.66
13	−1218.74	−374.08	−837.00	−7.66

S3.2.2 Coverage-dependence

Tables S11 and S12 show respectively the DFT and Hartree–Fock (HF) contributions, and RPA contribution to the MgO-CO adsorption energy at different coverages. Not only the RPA, but also the PBE and HF contributions are relatively sensitive to the coverage. The TZ2P results have been calculated using the T2 auxiliary basis set. Since we have previously seen that the error of the auxiliary fit in this case is $\Delta_{fit} = 0.14$ kcal/mol, we correct all TZ2P RPA contributions to the adsorption energy by this value, which results in the numbers shown in the last column of Table S12.

Table S11: Absolute energy components for different coverages Θ. PBE bonding energies (the energies of the total systems minus the energies of the atomic fragments) E_b^{PBE}, EXX energies $E_x@PBE$ evaluated with PBE orbitals, and the XC contribution $E_{xc}^{PBE}@PBE$ to the PBE bond energy. All values are in kcal/mol and have been evaluated using the TZ2P basis set.

	E_b^{PBE}			$E_x@PBE$			$E_{xc}^{PBE}@PBE$		
Θ	MgO-CO	CO	MgO	MgO-CO	CO	MgO	MgO-CO	CO	MgO
100	-868.68	-339.27	-526.02	-38935.19	-8348.64	-30583.84	-873.38	-268.14	-592.45
50	-1398.38	-343.43	-1051.96	-69486.31	-8336.81	-61143.98	-1456.39	-255.87	-1184.86
25	-2450.47	-343.37	-2103.92	-130629.43	-8336.85	-122286.93	-2640.90	-255.54	-2369.77

Table S12: RPA energy components in kcal/mol for different coverages Θ, and the corresponding contributions to the adsorption energies, evaluated with DZP and TZ2P basis sets.

Θ	Basis	$\sqrt{N_{\boldsymbol{k}}}$	MgO-CO	MgO	CO	E_c^{ads}	$E_c^{ads} + \Delta_{fit}$
100	TZ2P	11	−1218.62	−374.07	−836.87	−7.67	−7.53
50	TZ2P	9	−2059.30	−369.85	−1682.40	−6.97	−6.83
50	TZ2P	11	−2059.38	−369.86	−1682.50	−6.98	−6.84
100	DZP	11	−1033.21	−316.23	−710.38	−6.60	−
50	DZP	9	−1741.80	−312.17	−1423.73	−5.90	−
25	DZP	5	−3162.29	−311.37	−2845.19	−5.73	−
25	DZP	7	−3162.95	−311.40	−2845.82	−5.74	−

We did not explicitly calculate the RPA contribution to the adsorption energy at 25 % coverage with the TZ2P basis set. Instead, we note that when going from 100 % to 50 % coverage, the RPA contribution decreases by exactly 0.7 kcal/mol for both the DZP and TZ2P basis sets. This suggests that the coverage dependence of this quantity is essentially basis-set independent.

Based on this observation, we estimate the RPA contribution at 25 % coverage with the TZ2P basis set by correcting the corresponding 50 % value. Specifically, starting from the 50 % TZ2P result of −6.84 kcal/mol (already corrected for the auxiliary fit error of 0.14 kcal/mol), we apply the difference observed for the DZP basis set between 50 % and

25 % coverage ($-5.90 + 5.74 = 0.16$ kcal/mol). This yields

$$E_c^{\text{ads}}(\text{TZ2P}, \Theta = 25\%) = -6.68 \text{ kcal/mol}.$$

This value is used in the evaluation of the adsorption energy reported in Table 5 of the main text.

S3.3 Convergence with the number of MgO layers

Table S13: Absolute RPA@PBE energies for MgO, CO, and the corresponding dimer as well as the corresponding contribution to the adsorption energy for different numbers of MgO layers, calculated with the DZP basis set and the T2 auxiliary basis set. All values are in kcal/mol.

N_{layers}	MgO-CO	CO	MgO	E_c^{ads}
2	−1033.21	−316.23	−710.38	−6.60
3	−1392.23	−316.23	−1069.40	−6.60
4	−1751.42	−316.24	−1428.58	−6.60

S3.4 SCF energies for CO adsorption on the 4-layer MgO slab

Table S14: PBE bonding energies (the energies of the total systems minus the energies of the atomic fragments) E_b^{PBE}, EXX energies $E_x@PBE$ evaluated with PBE orbitals, and the XC contribution $E_{xc}^{PBE}@PBE$ to the PBE bond energy. All values are in kcal/mol and have been evaluated using the QZ4P basis set for a 4 MgO-layer slab.

	MgO-CO	CO	MgO
E_b^{PBE}	−1417.96	−339.88	−1074.59
$E_x@PBE$	−1535.59	−267.94	−1254.49
$E_{xc}^{PBE}@PBE$	−75 541.07	−9223.52	−66 314.54

References

(1) Spadetto, E.; Philipsen, P. H. T.; Förster, A.; Visscher, L. Toward Pair Atomic Density Fitting for Correlation Energies with Benchmark Accuracy. *Journal of Chemical Theory*

and Computation **2023**, *19*, 1499–1516.

(2) Lenthe, E. V.; Baerends, J. E. Optimized Slater-type basis sets for the elements 1–118. *Journal of Computational Chemistry* **2003**, *24*, 1142–1156.

(3) Harl, J.; Kresse, G. Cohesive energy curves for noble gas solids calculated by adiabatic connection fluctuation-dissipation theory. *Physical Review B* **2008**, *77*, 045136.

(4) Ren, X.; Merz, F.; Jiang, H.; Yao, Y.; Rampp, M.; Lederer, H.; Blum, V.; Scheffler, M. All-electron periodic G0W0 implementation with numerical atomic orbital basis functions: Algorithm and benchmarks. *Physical Review Materials* **2021**, *5*, 013807.
